# Information aggregation based trend prediction of energy structure via an improved compositional data time series forecasting model and its application

**DOI:** 10.1371/journal.pone.0351310

**Published:** 2026-06-26

**Authors:** Jingjing Ma

**Affiliations:** School of Finance and Accounting, Anhui Institute of International Business, Hefei, Anhui, China; Dr Shakuntala Misra National Rehabilitation University, INDIA

## Abstract

To improve the prediction accuracy of compositional data time series (CDTSs), the aggregation of compositional data was considered and applied to construct a combination forecasting model. Different from current arithmetic mean based aggregation of compositional data, the aggregation method of compositional data from the induced ordered weighted averaging (IOWA) operator was put forward. Properties of such aggregation methods are discussed. Since prediction accuracies of different individual forecasting models are diverse over time, forecasting error between the aggregated CDTSs and the original CDTS is minimized and set as an objective function of the aggregated weights. To derive the optimal weights associated to individual forecasting models, the genetic algorithm was utilized. Correspondingly, an improved time-varying combination mode and an IOWA operator based combination mode are developed. Finally, a numerical study on China’s primary energy production structure is presented. The results show that the developed varying weight combination model is superior to the benchmark model in terms of prediction accuracy comparison, illustrating the feasibility and validity of the developed combination forecasting model.

## 1. Introduction

Generally, primary energy includes non renewable energy (e.g., fossil fuels, nuclear energy, hydropower, wind energy, solar energy) and renewable energy (e.g., biomass energy). To describe the development and evolution of regional energy, the energy structure is a key object. Currently, the consumption of fossil fuels still dominates China’s energy consumption. Emerging energies include nuclear energy, hydropower, wind energy and solar energy have achieved sustained development, while the proportion still needs to be further increased for the sake of carbon peaking and carbon neutrality goals. Forecasting regional energy structure is of significant importance.

As one of the world’s largest energy producer and energy consumer, rich coal, poor oil and low gas are three main characteristics of China’s energy resources. Coal is the main energy source for China’s energy production and consumption. From the perspective of world energy’s development trends, the development and utilization of new and renewable energy become an important direction. To realize a clear understanding about the future trend, how to provide an accurate forecasting on the primary energy production structure could provide guiding conclusions for future energy development plan.

**Fig 1:**
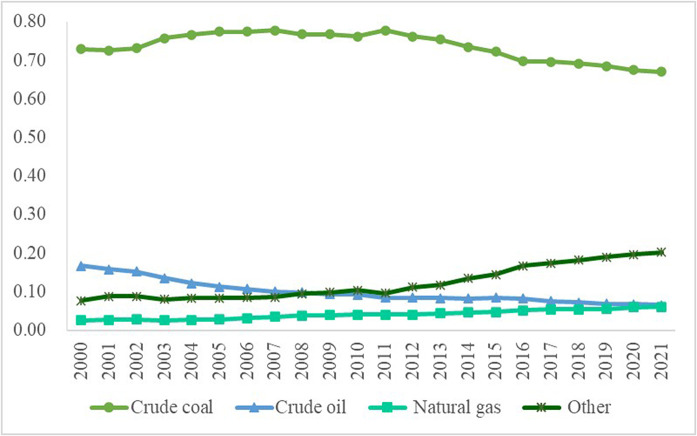
Annual data of primary energy production structure in China from 2000 to 2021.

Fig 1 shows the annual data of primary energy production structure in China during the period of 2000–2021 (Data source: http://www.stats.gov.cn/). Totally, it can be concluded that the percentage of crude coal has be decreased in the past two decades. On the other hand, it’s still the main energy source in China. Besides, crude oil owns a similar trend. Different from the two sources mentioned above, percentages corresponding to the natural gas and the other energy sources perform a increasing trend.

To analyze the energy production structure, Hou and Song [1] considered the optimization of energy structure in China. The measures include adopting energy price or carbon tax are suggested. Liu et al. [2] analyzed the influences of consuming coal, oil, natural gas, hydropower, solar and wind energy, nuclear power, and other renewable energy sources on carbon emissions in European Union. By using a dynamic stochastic general equilibrium model, Jin et al. [3] quantified the macroeconomic effects of an unconventional monetary policy–targeted refinancing, and a conventional monetary policy–general refinancing on the optimization of the energy supply structure. Li et al. [4] introduced the Markov chain to set up an improved energy structure prediction model. Vorontsova et al. [5] considered the innovative energy producing structures in Russia. Currently, most of the researchers mainly pay attention on the energy consumption structure [6,7,8,9,10,11], while forecasting on the energy producing structure was not widely discussed.

To forecast observations with different components and corresponding proportional information, the concept of compositional data comes to be a valid tool [12]. Since compositional data owns the characteristic of constant sum to 1 constraint, it can reflect many scenarios, which has been widely utilized to predict real-world events [13–16]. Current studies on CDTSs mainly focused on the processing of compositional data, forecasting on CDTSs relies on common forecasting models and the inverse transformation corresponding to the transformed data. It’s worth noting that combination forecasting has shown to be a valid tool to improve forecasting accuracy [17–19]. Besides, since the time-varying weights of combination forecasting can also be seen as a CDTSs, the integration of combination forecasting and compositional time series could provide a novel forecasting mode.

By reviewing the history of existed publications, the following research gaps can be summarized: 1) To make a scientific and reasonable energy plan, forecasting of energy producing structure is needful. Knowledge about the energy producing structure would be useful to improve the reasonability of possible energy plan. 2) From the theoretical perspective, how to design a CDTSs forecasting model with high accuracy is still an open and interesting problem.

In this paper, the combination forecasting model is chosen for the forecasting of energy producing structure presented by CDTSs. Relying on individual forecasting results, it is common to obtain prediction results that are closer to actual observation data by integrating individual results. As an extension of aggregation model, the induced ordered weighted averaging (IOWA) operator [20] was chosen to aggregate individual predicted CDTSs.

## 2. Literature review

With the background mentioned above, this section mainly summarizes current studies on compositional data and corresponding time series analysis, combination forecasting and IOWA operator.

### 2.1. Compositional time series analysis

To describe the observed data with proportional information, compositional data [21,22] provides a valid tool. For instance, the energy producing structure [7, 23], the energy consumption structure [8, 9, 10, 24] and market share [14]. Thus, compositional data time series (CDTS) is the sequence that the observed data in each time point is a compositional data, i.e., $CTS=\{(\overset{t}{\underset{1}{x}},\overset{t}{\underset{2}{x}},\cdots ,\overset{t}{\underset{p}{x}})|t=1,2,\cdots ,T\}$, where *p* is the dimension and *T* is the sample size.

For the sake of forecasting compositional time series, data preprocessing is first considered. Up until now, typical data preprocessing models include logarithm transformation [21] and hyperspherical transformation [25]. The logarithm transformation models mainly include asymmetric logarithmic ratio transformation (ALRT) and central logarithmic ratio transformation (CLRT). On the other hand, the scenario with 0 and 1 as components cannot be handled. Hence, Wang et al. [25] developed the hyperspherical transformation model, which can deal with the scenario with 0 as component(s). The application areas of compositional data were then enlarged. So far, diverse forecasting methods for CDTSs have been developed. Petra et al. [13] considered the generalization of vector autoregressive models. Snyder et al. [14] developed a state space approach. Zhou et al. [26] put forward the autoregressive Dirichlet estimation method. The usage of VARIMA model was extended by Carles et al. [27]. Chang et al. [28] proposed a new class of seasonal time series models based on a stable seasonal composition assumption. It can be seen that classical statistical forecasting models have been widely generalized under the environment of CDTSs.

### 2.2. Combination forecasting

To improve the forecasting accuracy, Bates and Granger [29] introduced the concept of combination forecasting. By weighting the results produced by individual forecasting models, the combination forecasting result is commonly more closer to the real observations. Since then, the combination forecasting has been widely studied in theory and applied to real-world applications. Liu et al. [30] proposed a combination forecasting model based on the hybrid interval multi-scale decomposition method, which is applied to forecasting interval-valued carbon prices. Radchenko et al. [31] provided the first thorough investigation of the negative weights in combination forecasting. Qian et al. [17] proposed an AI-AFTER method, which can determine the appropriate goal of forecast combination. Besides, proper goal of the combination forecasting can be automatically achieved. Kang et al. [18] suggested a change of focus from the historical data to the produced forecasts, so that the features in forecast combinations can be extracted. Following the combination forecasting mode given by Bates and Granger [29], the essence of combination forecasting is data fusion, which are commonly presented by type of mean values [19].

Currently, in the field of CDTSs forecasting, the combination forecasting mode has not yet received sufficient attention. Besides, applications of the fusion of compositional data has also merely been discussed [32]. Furthermore, current information aggregation of compositional data was designed by using fundamental mathematical means, which cannot be used for complex scenarios. Since the induced ordered weighted averaging operator (IOWA for short) [20] is a generalization of types of mathematical means include common weighted arithmetic mean. IOWA has also been applied to build combination forecasting model [33]. Hence, the IOWA is chosen as further tool for the fusion of individual forecasting CDTSs.

Actually, the application of aggregation operators in combination forecasting has been reported [34,35]. By weighting different individual forecasting models according to the accuracies of these models at different time points, strengths of the individual forecasting model with larger accuracies could be enlarged. Hence, the combination forecasting of CDTSs from the perspective of information aggregation could be considered [36,37].

### 2.3. Aim, contribution and organization

Due to the literature review and analysis mentioned above, the aim of this paper is to consider a combination forecasting model for CDTSs from the perspective of information aggregation. To realize the motivation, the contributions of this paper can be summarized as below:

1) Theoretically, a novel compositional data aggregation process is discussed. Pérez-Fernández et al. [32] considered a general betweenness-based aggregation framework for compositional data. Although theoretical studies on the general betweenness-based aggregation framework has been developed, detailed compositional data aggregation functions need to be further detailed. We integrated the concept of induced ordered weighted averaging operator and compositional data, a novel compositional data aggregation model is provided. Properties of the developed novel compositional data aggregation functions still need to be analyzed.
2) From the methodological perspective, the combination forecasting model of CDTSs is developed. Currently, how to improve the prediction accuracy of CDTSs is an open problem. To enlarge the strengths of individual forecasting model at different time points, a time-varying weighted and IOWA based combination forecasting structure of CDTSs are put forward in this paper.
3) Technologically, for the out-of-sample forecasting of CDTSs, a bilayer forecasting procedure is developed, i.e., the forecasting of time-varying weighting vectors and the forecasting of individual CDTSs models. By setting the weighting vectors as a CDTS, the problem of out-of-sample time-varying combination forecasting can be solved.

The rest of this paper is structured as follows: Fundamental notions, notations and the main results are shown in the Main Results section. Application of the developed combination forecasting model for CDTS in the field of energy producing structure in China is shown in the Numerical Study section. The Conclusion section summarizes the conclusions and possible future work.

## 3. Main results

### 3.1. Induced ordered weighted compositional data aggregation function

From the perspective of mathematics, a compositional data is presented as a *p*-dimension vector, denoted as $X=(x_{1},x_{2},\cdots ,x_{p}{)}^{T}$, where $0\leq x_{j}\leq 1,j=1,2,\cdots ,p$ and $\sum_{j=1}^{p}x_{j}=1$. Since the compositional data satisfies the characteristic of sum-constrained to 1, catastrophic consequences would produced if the characteristic was intentionally or unintentionally ignored. Thus, Aitchison [38] introduced the logratio transformation to realize the analysis of compositional data. Since then, another logratio transformation methods include centered logratio transformation and isometric logratio transformation were developed. On the other hand, the logratio transformation models are limited under the scenarios with 0 or 1 as component. Wang et al. [25] introduced the hyperspherical transformation model, i.e.,

For a given compositional data $x=\left(x_{1},x_{2},\ldots ,x_{p}\right)$, let $z_{i}=\sqrt{x_{i}},i=1,2,\cdots ,p$, then $\overset{2}{\underset{1}{z}}+\overset{2}{\underset{2}{z}}+\cdots +\overset{2}{\underset{p}{z}}=1$. Then, the vector $Z=\left(z_{1},z_{2},\cdots ,z_{p}\right)$ can be regarded as a point on the hypersphere. Then,


$$\begin{array}{c} \{\begin{array}{c} z_{1}=\sin\theta_{2}\sin\theta_{3}\sin\theta_{4}\cdots \sin\theta_{p}\\ z_{2}=\cos\theta_{2}\sin\theta_{3}\sin\theta_{4}\cdots \sin\theta_{p}\\ z_{3}=\cos\theta_{3}\sin\theta_{4}\cdots \sin\theta_{p}\\ \vdots \\ z_{p-2}=\cos\theta_{p-2}\sin\theta_{p-1}\sin\theta_{p}\\ z_{p-1}=\cos\theta_{p-1}\sin\theta_{p}\\ z_{p}=\cos\theta_{p} \end{array} \end{array}$$ (1)

By inverting Eq. (1), it can be obtained that:


$$\begin{array}{c} \{\begin{array}{c} \theta_{p}=\arccos z_{p}\\ \theta_{p-1}=\arccos\left(\frac{z_{p-1}}{\sin\theta_{p}}\right)\\ \theta_{p-2}=\arccos\left(\frac{z_{p-2}}{\sin\theta_{p}\sin\theta_{p-1}}\right)\\ \vdots \\ \theta_{2}=\arccos\left(\frac{z_{2}}{\sin\theta_{p}\sin\theta_{p-1}\cdots \sin\theta_{3}}\right) \end{array} \end{array}$$ (2)

It can be seen that the scenario with 0 as component(s) can be handled by the hyperspherical transformation model. By Eq. (2), initial compositional data is transformed with *p* dimensions to be p−1 series of θ. When using logarithmic transformation method, the range of p-1 sequences after hyperspherical transformation is smaller and therefore more stable. As a result, it would be beneficial for accurate prediction.

To aggregate compositional data, Pérez-Fernández et al. [32] introduced the betweenness-based aggregation mode [39], which can be defined in the following:

**Definition 2.1**. Let (*X*, *B*, *S*) be a bounded beset and n∈ℕ, a function $A:X^{n}\to X$ is named as an (*n*-ary) aggregation function on (*X*, *B*, *S*) if

(1) it satisfies the boundary conditions, i.e., A(o,o,⋯,o)=o for any o∈S;
(2) it is monotone, i.e., for any o∈S and any $x=(x^{\left(1\right)},x^{\left(2\right)},\cdots ,x^{\left(n\right)})$, $y=(y^{\left(1\right)},y^{\left(2\right)},\cdots ,y^{\left(n\right)})\in X^{n}$, the fact that $(o,x^{\left(i\right)},y^{\left(i\right)})\in B$ for any i∈{1,2,⋯,n} implies that (A(o),A(x),A(y))∈B, where o=(o,o,⋯,o).

Herein, the concepts of betweenness relation *B* and beset (*X*, *B*) can be founded in Pérez-Fernández et al. [39].

Given that $x^{\left(1\right)},\cdots ,x^{\left(n\right)}\in S_{k}$ are *n* compositional data vectors and $w=(w_{1},\cdots ,w_{n})$ is a suitable weighing vector satisfying $\sum_{i=1}^{n}w_{i}=1$, Pérez-Fernández et al. [39] introduced the following natural aggregation function, i.e.,


$$\begin{array}{c} C_{w}\left(x^{\left(1\right)},\ldots ,x^{\left(n\right)}\right)(j)=\sum_{i=1}^{n}w_{i}x^{\left(i\right)}(j), \end{array}$$ (3)

for any j∈{1,⋯,k}.

With the concept of IOWA operator, we have

**Definition 2.2**. Let $\left(u_{1},x^{\left(1\right)}),\cdots ,(u_{n},x^{\left(n\right)}\right)$ be *n* 2-tuple compositional data arrays composed by *n* compositional data vectors $x^{\left(1\right)},\cdots ,x^{\left(n\right)}\in S_{k}$ and corresponding induced values $u_{1},\cdots ,u_{n}$, the induced ordered weighted compositional data aggregation function, denoted as $\overset{IOWA}{\underset{w}{C}}:(S_{k}{)}^{n}\to S_{k}$, can be defined according to,


$$\begin{array}{c} \overset{IOWA}{\underset{w}{C}}\left((u_{1},x^{\left(1\right)}),\ldots ,(u_{n},x^{\left(n\right)})\right)(j)=\sum_{i=1}^{n}w_{i}x^{\left(\sigma (i)\right)}(j), \end{array}$$ (4)

where $x^{\left(\sigma (i)\right)}$ is the compositional data vector corresponding to the *i*-th largest induced value $u_{\sigma (i)},i=1,2,\cdots ,n$.

Herein, the induced values are commonly numerical values that represent the importance of associated compositional data.

According to Eq. (4), the following results can be derived from the perspective of compositional data aggregation.

**Property 1**. The induced ordered weighted compositional data mean defined by Eq. (4) is a valid betweenness-based aggregation function.

**Proof**. Firstly, we prove that $\overset{IOWA}{\underset{w}{C}}$ satisfies the boundary conditions.

Let $o=(o_{1},o_{2},\cdots ,o_{n})$ be any element in ***S***, then if $x^{\left(i\right)}=o,i=1,2,\cdots ,m$, i.e., $x^{\left(i\right)}(j)=o_{j}$, we have


$$\begin{array}{c} \overset{IOWA}{\underset{w}{C}}\left((u_{1},x^{\left(1\right)}),\ldots ,(u_{n},x^{\left(n\right)})\right)(j)=\sum_{i=1}^{n}w_{i}x^{\left(\sigma (i)\right)}(j)=\sum_{i=1}^{n}w_{i}o_{j}=o_{j}. \end{array}$$

Thus, it can be derived that the property of boundary conditions are valid.

Secondly, with given associated induced values, denoted as $\left(u_{1},u_{2},\cdots ,u_{n}\right)$, the proof of monotonicity is similar to Proposition 7 in Pérez-Fernández et al. [39]. Thus, the proof is omitted.☐

**Property 2**. The aggregated result produced by Eq. (4) is also a compositional data.

**Proof**. By Eq. (4), it can be seen that the induced ordered weighted compositional data aggregation function is a weighted averaging mean in essence.

For the summarization of the aggregated compositional data, denoted as $v_{sum}$, the following result is valid,


$$\begin{array}{c} v_{sum}=\sum_{j=1}^{k}\sum_{i=1}^{n}w_{i}x^{\left(\sigma (i)\right)}(j)=\sum_{i=1}^{n}w_{i}\sum_{j=1}^{k}x^{\left(\sigma (i)\right)}(j)=\sum_{i=1}^{n}w_{i}=1. \end{array}$$

Thus, it’s proved that the aggregated result produced by Eq. (4) is a compositional data.☐

**Property 3**. When $u_{1}\geq u_{2}\geq \cdots \geq u_{n}$, the induced ordered weighted compositional data aggregation function is degenerated to common aggregation function of compositional data vectors.

### 3.2. Compositional data time series combination forecasting: From the perspective of data aggregation

**Fig 2:**
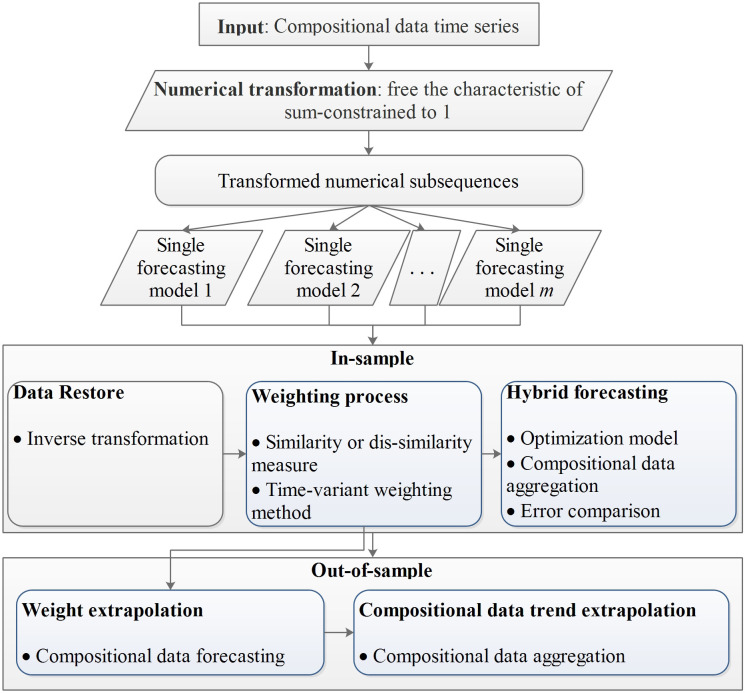
Hybrid forecasting structure for compositional data time series.

In this subsection, a hybrid forecasting structure for CDTSs from the perspective of information aggregation is developed. The forecasting procedure is shown in Fig 2.

By Fig 2, a time-varying combination forecasting procedure is developed. The weighting vectors are also seen as a CDTSs so that the out-of-sample forecasting on the observed CDTSs can be realized. Next, the total hybrid forecasting procedure is illustrated in detail.

#### 3.2.1. Determination of weighting vectors.

Let $X=\{(\overset{t}{\underset{1}{x}},\overset{t}{\underset{2}{x}},\cdots ,\overset{t}{\underset{p}{x}}),t=1,2,\cdots ,n\}$ be a compositional data time series. Assume that ${\hat{X}}_{k}=\{(\overset{t}{\underset{k1}{\hat{x}}},\overset{t}{\underset{k2}{\hat{x}}},\cdots ,\overset{t}{\underset{kp}{\hat{x}}}),t=1,2,\cdots ,n;k=1,2,\cdots ,K\}$ are the predicted compositional data time series given by *K* individual forecasting models. To realize the combination forecasting of CDTSs, the weighting mode is firstly introduced.

*Time invariant weighting mode*. In this mode, individual forecasting results are aggregated with a time-invariant weighting vector. Given that $\Omega =\{(\omega_{1},\omega_{2},\cdots ,\omega_{K})\}$ is the time-invariant weighting vector corresponding to the individual forecasting models, while $f_{\Omega }$ is an aggregation function with Ω. Thus, the combined forecasting results ${\hat{X}}^{CF}$ corresponding to ***X*** can be obtained according to


$$\begin{array}{c} {\hat{X}}^{CF}=f_{\Omega }({\hat{X}}_{1},{\hat{X}}_{2},\cdots ,{\hat{X}}_{K}). \end{array}$$ (5)

To derive the time-invariant weighting vector Ω, the following optimization model is commonly used:


$$\begin{array}{c} \begin{array}{c} \;\min z=dissim\left(X,{\hat{X}}^{CF}\right)\\ or\\ \;\max z=sim\left(X,{\hat{X}}^{CF}\right)\\ \;s.t.,\left\{\begin{array}{c} \sum_{k=1}^{K}\omega_{k}=1,\\ \omega_{k}\in [0,1], \end{array}\right\}. \end{array} \end{array}$$ (6)

where dissim(*,*) and sim(*,*) are respectively the dissimilarity and similarity measures between any two compositional data time series.

The optimization model provides an objective way to derive the weighting vector. It’s worth noting that there are some another non-optimal weighting methods including entropy weights and equal weights processing. Since the optimization model can realize given combination forecasting efficiency like minimizing the sum of squares of errors, the optimization model is selected.

*Time-varying weighting mode.* In this mode, individual forecasting models are weighted with associated time-varying weighting vector, i.e., the weighting vectors are different with the changing of time. Let $\Omega_{t}=(\overset{t}{\underset{1}{\omega }},\overset{t}{\underset{2}{\omega }},\cdots ,\overset{t}{\underset{K}{\omega }}),t=1,2,\cdots ,n$ be the weighting vector corresponding to the *t*-th time point. Thus, *t*he combined forecasting compositional data at the *t*-th time point, deno*t*ed as $\overset{CF}{\underset{t}{\hat{X}}}$ can be derived according to


$$\begin{array}{c} \overset{CF}{\underset{t}{\hat{X}}}=f_{\Omega_{t}}({\hat{X}}_{1},{\hat{X}}_{2},\cdots ,{\hat{X}}_{K}). \end{array}$$ (7)

Correspondingly, determination of the time-varying weighting vectors can be obtained by the following optimization model:


$$\begin{array}{c} \begin{array}{c} \;\min z=\sum_{t=1}^{N}dissim\left(X_{t},\overset{CF}{\underset{t}{\hat{X}}}\right)\\ or\\ \;\max z=\sum_{t=1}^{N}sim\left(X_{t},\overset{CF}{\underset{t}{\hat{X}}}\right)\\ \;s.t.,\left\{ \begin{array}{c} \sum_{k=1}^{K}\overset{t}{\underset{k}{\omega }}=1,t=1,2,\cdots ,K,\\ \overset{t}{\underset{k}{\omega }}\in [0,1],t=1,2,\cdots ,K. \end{array}\right. \end{array} \end{array}$$ (8)

To obtain the optimal time-varying weighting vectors, genetic algorithm is utilized [40]. The main idea of the algorithm is to introduce the parameters to be optimized into the gene coding, and then construct an adaptation function according to the objective function. Through the genetic operations such as selection, crossover and mutation, the algorithm is continuously optimized until the optimal solution is obtained.

The process of genetic algorithm to seek the optimal time-varying weighting vectors is summarized as follows:

(1) Population initialization: An initial population containing *K* individuals is randomly generated. Each chromosome contains *n* genes $w=\left[w_{1},w_{2},\cdots ,w_{n}\right]$, where *n* is the number of weight. The random numbers are randomly selected in [0,1] as the initial weight.
(2) Coding: Binary coding is adopted in this paper to encode each chromosome, and each individual is composed of binary strings.
(3) Construct the fitness function: The optimization obtained by genetic algorithm is the maximum fitness. However, the objective function in the developed model is to minimize the error. The fitness function is thus set as the opposite number of the objective function.
(4) Selection: Select individuals to form a new group according to a certain probability. The probability of individuals being selected is defined as:

$$\begin{array}{c} P_{i}=\frac{F(w_{i})}{\sum_{i=1}^{K}F(w_{i})}, \end{array}$$ (9)

where $F(w_{i})$ is the fitness of the *i*-th individual.

(5) Genetic operators: Crossover operators and mutation operators are utilized.

The crossover operation is to select two individuals and exchange genes at corresponding positions according to the crossover probability $P_{c}$. The mutation operation is to select an individual in the population and change the gene in the corresponding position according to the mutation probability $P_{m}$.

(6) Set the number of iterations and repeat steps (3)-(5) until the end of the iteration.

As a population-based stochastic search method, genetic algorithm excels at navigating the complex, potentially non-convex landscape of weight space, especially when the objective function is discontinuous, noisy, or lacks closed-form gradients. Such method naturally respects the simplex constraints through tailored encoding (e.g., normalizing candidate weights to sum to 1) and does not rely on gradient information, making them robust to ill-behaved objective functions.

#### 3.2.2. Construction of the combination forecasting.

As shown in 5 and 7, hybrid CDTSs forecasting can be realized by using the developed compositional data aggregation functions.

Next, let *f* be the natural aggregation function or the IOWA operator mentioned in subsection 3.1, the hybrid forecasting of CDTSs will be analyzed both within and outside the sample period.

For the time-invariant mode within the sample period, the hybrid fitted compositional data sequence can be directly derived according to Eq. 5.

For the time-invariant mode outside the sample period, since the fixed weighting vector is still valid, the hybrid forecasted compositional data sequence can also be directly derived according to Eq. 5.

For the time-varying mode within the sample period, the hybrid fitted compositional data sequence can be directly derived according to Eq. 7.

Herein, the weights within the sample period can be obtained according to Eq. 8. Since the time-invariant weighting vector can also be seen as a compositional data. To derive the out-of-sample weights, the weighting vector is simultaneously predicted by the developed forecasting model.

The combinational forecasting procedure can be summarized as below:

**Algorithm 1: Combination forecasting of CDTSs**

1: Input: Observed CDTSs

2: Output: Predicted CDTSs

3: Start

4: Transform observed CDTSs by using Eq. (2)

5: Forecast the transformed multiple sequences by using individual forecasting models

6: Transform individual predicted results by inverse transformation model

7: Calculate time-invariant weights and time-varying weights by Eq. (8)

8: Obtain the combination forecasting results by Eq. (5) or Eq. (7)

9: Compare different forecasting models by error indicators

10: The end

For the time-varying mode outside the sample period, the out-of-sample predictions need to further determine the time-varying weighting vectors. The reason is that the actual compositional data are missing during this period so the weighting vectors cannot be produced. On the other hand, it’s worth noting that the weighting vectors also satisfy the characteristic of sum-constraint to 1. As a result, the time-varying weighting vectors are collected and set as a CDTSs. The out-of-sample time-varying weighting vectors can thus been predicted. Furthermore, the vectors can be utilized to drive the out-of-sample combined compositional data predictions with the forecasting of individual out-of-sample compositional data predictions.

## 4. Numerical study

### 4.1. Evaluation and comparison on the developed compositional data time series analysis model

Except traditional prediction error indexes include RMSPE, MAPE, et al., the prediction error corresponding to compositional data time series is often measured by the Aitchsion distance in the uniform space. The formula for the norm and the distance between two compositional data units in the simplex space is defined as:


$$\begin{array}{c} ∥x\overset{2}{\underset{S}{∥}}={\left\langle x,x\right\rangle }_{S}=\sum_{i=1}^{p}{\left(\log\frac{x_{i}}{g(x)}\right)}^{2} \end{array}$$ (10)

$$\begin{array}{c} d_{S}\left(x,y\right)=\sqrt{\sum_{i=1}^{p}{\left(\log\frac{x_{i}}{g\left(x\right)}-\log\frac{y_{i}}{g\left(y\right)}\right)}^{2}} \end{array}$$ (11)

where $g(x)=(\overset{p}{\underset{i=1}{\Pi }}x_{i}{)}^{1/d}$.

To comprehensively show the superiority of the developed model, two traditional error evaluation indexes RMSPE and MAPE and four evaluation indexes MSD, SSD, MCPE and CVPE are selected. Table 1 shows the error indicators used in this manuscript.

**Table 1:** Error indicators for evaluation.

Error Index	Abbreviation	Formulation
Root-mean-squares percentage error	RMSPE	$\sqrt{\frac{1}{N}\sum_{i=1}^{N}\sum_{j=1}^{D}{\left(\frac{y_{ij}-{\hat{y}}_{ij}}{y_{ij}}\right)}^{2}}\times 100\%$
Mean absolute percentage error	MAPE	$\frac{1}{N}\sum_{i=1}^{N}\sum_{j=1}^{D}\frac{\left|y_{ij}-{\hat{y}}_{ij}\right|}{\left|y_{ij}\right|}\times 100\%$
Mean distance error	MSD	$\frac{1}{N}\sum_{i=1}^{N}d_{S}\left(y_{i},{\hat{y}}_{i}\right)$
Sum of squares of distances	SSD	$\sum_{i=1}^{N}d_{S}{\left(y_{i},{\hat{y}}_{i}\right)}^{2}$
Mean component percentage error	MCPE	$\frac{1}{N}\sum_{i=1}^{N}\frac{d_{S}{\left(y_{i},{\hat{y}}_{i}\right)}^{2}}{||y_{i}|\overset{2}{\underset{S}{|}}}\times 100\%$
Component vector percentage error	CVPE	$\frac{\sum_{i=1}^{N}d_{S}{\left(y_{i},{\hat{y}}_{i}\right)}^{2}}{\sum_{i=1}^{N}||y_{i}|\overset{2}{\underset{S}{|}}}\times 100\%$

### 4.2. Experimental results

In this subsection, three individual forecasting models include Holt’s Linear Trend Method (HLTM), Support Vector Regression (SVR) and Extreme Learning Machine (ELM) were used to model training the training samples (Main codes can be seen in the supporting information S1 File), i.e.,

HLTM: The scenario that the observed time series data with linear trends but no seasonality is applicable. HLTM adds trend estimation on the basis of simple exponential smoothing, which smooths the level and trend separately through two equations, i.e.,


$$\begin{array}{c} \left\{ \;\;\;\begin{array}{c} Level:\;L_{t}=\alpha A_{t}+\left(1-\alpha \right)\left(L_{t-1}+T_{t-1}\right),\\ Trend:\;T_{t}=\beta \left(L_{t}-L_{t-1}\right)+\left(1-\beta \right)T_{t-1},\\ Forecast:\;F_{t+m}=L_{t}+mT_{t}. \end{array}\right. \end{array}$$

HLTM is suitable for mid-term forecasting and can capture linear trends.

SVR: A classic variation of Support Vector Machine (SVM) in regression problems. The core issue is to find the optimal fitting hyperplane while allowing for controllable errors. Both nonlinear modeling capabilities and anti overfitting properties are included. Different from linear regression and decision tree regression, SVR does not pursue perfect fitting of all data points, but focuses on key support vectors, making it suitable for handling numerical prediction problems with small and medium-sized datasets and complex nonlinear relationships.

ELM: ELM is a learning algorithm used for single hidden layer feedforward neural networks. The characteristic of this model is that the input weights and bias terms are randomly assigned, and the training process can be completed by adjusting the output weights. Such characteristic gives ELM extremely high computational efficiency and good generalization ability.

Given that $\theta_{2}$, $\theta_{3}$ and $\theta_{4}$ are transformed series of the initial observed CDTSs. In this paper, the data from first, second and third order lags were taken as independent variables for SVR and ELM. The radial basis function was chosen as the kernel function for SVR, with the penalty factor and kernel parameter set to the default values of R Language (1 and 0.333). The activation function for ELM was selected as the sigmoid function. The step size was set to 1 to increase the number of hidden neurons for training the model. The results indicated that the best result was achieved when the number of hidden neurons was 6, 5, and 6 for predicting $\theta_{2}$, $\theta_{3}$ and $\theta_{4}$, respectively.

Based on the results of three individual models, the proposed optimization model is solved by using the genetic algorithm. Correspondingly, varying weights in combination model on the training set can be obtained, which are presented in Table 2. To determine the varying combination weights on the testing set, the method of compositional data prediction is employed. This involves treating each group of variable weight weights on the training period as a CDTS. By performing spherical coordinate transformation, the Holt exponential smoothing method is used to predict the weights’ CDTS. Varying weights used in testing data set are then obtained by performing inverse transformation on the predicted results.The population size, crossover rate and mutation rate of GA are respectively set as 20, 0.6 and 0.001. The results are also displayed in Table 2. Additionally, the time-invariant optimal weights obtained using the IOWA operator are *w*_1_ = 0.7526, *w*_2_ = 0.1482, *w*_3_ = 0.0992.

**Table 2:** Weights information of varying weight combination model.

Data	Year	*w*_1_	*w*_2_	*w*_3_
training set	2003	0.4218	0.3808	0.1974
	...	...	...	...
	2016	0.0153	0.2691	0.7156
	2017	0.435	0.4164	0.1486
testing set	2018	0.2780	0.3529	0.3691
	2019	0.2873	0.3417	0.3710
	2020	0.2966	0.3306	0.3728
	2021	0.3058	0.3195	0.3747

Table 3 shows the overall performance of five prediction methods. It is evident that the varying weight combination prediction and IOWA operator have yielded favorable results on the training set. Error indicators of the varying weight combination model are lower than those of the three individual forecasting models. The genetic algorithm based varying weight combination model outperforms traditional time-invariant combination model. IOWA operator based combination prediction results are totally lower than individual models and time-invariant weight combination model.

**Table 3:** Comparisons among different forecasting models.

Data	Model	RRMSE	MAPE	MSD	SSD	CVPE	MCPE
training set	Holt	0.0934	0.1449	0.0288	0.0144	0.0011	0.0011
	SVR	0.0903	0.1136	0.0270	0.0171	0.0013	0.0011
	ELM	0.0837	0.1079	0.0243	0.0129	0.0010	0.0009
	Invariable Weight	0.0813	0.1064	0.0241	0.0121	0.0009	0.0008
	Varying Weight	**0.0788**	**0.1051**	0.0235	0.0109	0.0008	0.0008
	IOWA	0.1309	0.2979	**0.0185**	**0.0081**	**0.0006**	**0.0006**
testing set	Holt	0.1363	0.1946	0.0498	0.0119	0.0041	0.0041
	SVR	0.4350	0.7645	0.1884	0.1566	0.0536	0.0540
	ELM	0.0997	0.1590	0.0364	0.0054	0.0019	0.0019
	Invariable Weight	0.1542	0.2491	0.0630	0.0176	0.0060	0.0061
	Varying Weight	0.0862	0.1258	0.0313	0.0046	0.0016	0.0016
	IOWA	**0.0634**	**0.1068**	**0.0246**	**0.0026**	**0.00309**	**0.0009**

On the training set (compositional datum located from 2003 to 2017), the genetic algorithm based varying weight combination model performs the best on RMSPE and MAPE. On the other hand, the IOWA operator based combination model performs better on MSD, SSD, CVPE, and MCPE. It achieved reductions of 0.50%, 0.29%, 0.02%, and 0.02% compared to the varying weight combination model. On the testing set (compositional datum located from 2018 to 2021), both the varying weight and IOWA operator based combination models significantly reduced prediction errors compared to individual models and the time-invariant weight combination model. Specifically, compared to the time-invariant weight combination model, the varying weight combination and IOWA operator based combination model reduced RMSPE by 1.35% and 3.62%, MAPE by 3.31% and 5.22%, MSD by 0.51% and 1.18%, and CVPE by 0.03% and 0.10%, respectively. Furthermore, the varing weight combination model outperformed the IOWA operator version in terms of prediction accuracy. This indicates that the varying weight combination and IOWA operator based combination model have higher prediction accuracies compared to other models.

**Fig 3:**
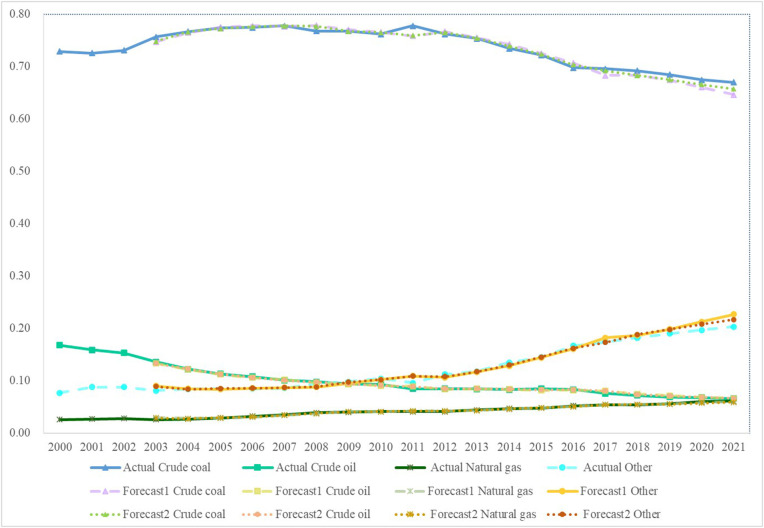
Actual and predicted values of primary energy production structure.

Fig 3 shows the comparison between the predicted and actual values of China’s Primary energy production structure using two combination methods. It is evident that the predicted values closely align with the actual values, suggesting that the proposed combination prediction method exhibits a strong predictive capability. The evolving trend of China’s Primary energy production structure from 2000 to 2021 can be effectively captured.

### 4.3. Discussion

By Table 3 and Fig 3, it can be seen that the combination forecasting results are nearly coincident with the original observations. Strengths of the developed combination forecasting model can be shown. The error level of test set is greater than training set, which is acceptable. Besides, the test period (20% of the observations) is short due to the size of total dataset is not large. On the other hand, since the accuracies of the forecasting results between the training dataset and the testing dataset locate at the same numerical level, there is no overfitting problem. Furthermore, the combination forecasting is realized by weighting the individual forecasting results. As a result, generalization ability of the developed combination forecasting model can be kept or even improved by the generalization ability of individual forecasting models.

From the trend perspective, traditional energy is gradually shifting from being the main base load energy to the role of peak shaving guarantee. Advanced coal production capacity is released in an orderly manner to ensure stable supply. The oil and gas sector continues to promote the increase of reserves and production. At the same time, natural gas, as the bridge of energy transformation, plays a key role in peak shaving guarantee, helping to ensure energy supply security and green transition. Besides, new energy has become the core driving force for optimizing the energy structure, and the proportion of non-fossil energy consumption continues to increase. The installed capacity of wind and photovoltaic power generation has surpassed that of thermal power for the first time, and the installed capacity of new energy storage remains the world’s largest scale. The total amount of hydropower, nuclear power, wind power, and solar power generation has significantly increased. The integration of artificial intelligence and the energy industry is accelerating, promoting the upgrading of energy production and consumption towards intelligence and efficiency. Emerging energy such as hydrogen energy has entered a critical stage of large-scale application, and clean energy technology is accelerating global sharing, reshaping the global energy supply chain. The predicted results show that current energy plan and policies are effective, which transforming the energy structure towards a green and better direction.

In the future, it’s suggested to promote the orderly transformation of traditional energy, release advanced coal production capacity to ensure stable supply, and strengthen the transformation of coal-fired power into a basic guarantee and flexible regulation function. By utilizing modern coal chemical technology to convert coal from fuel to raw materials and clean fuels, we aim to enhance oil and gas storage and production capacity to solidify our bottom line capability. At the same time, we should leverage natural gas as a bridge for energy transition to ensure the resilience of energy supply. Besides, new energy can be taken as the core driving force for structural optimization to expand the installed capacity of wind and photovoltaic power. it’s also suggested to focus on promoting the construction of large-scale bases and distributed and decentralized energy sources in the Shage Desert region, consolidating the global leading advantage in wind and solar power installed capacity surpassing thermal power and energy storage. Finally, nuclear power, coordinate hydropower development and ecological protection could be safely and orderly developed, so that the proportion of non fossil energy consumption can be increased, forming a multi energy complementary system.

## 5. Conclusion

In light of the limitations of existing combined models for forecasting CDTSs, novel combined forecasting methods based on genetic algorithm for varying weight shown by compositional data and IOWA operator are proposed. The effectiveness of these methods is verified by applying it to China’s Primary energy production structure. The data is divided into training and testing sets, and the weights are obtained by minimizing the error using genetic algorithms. Comparing the individual forecasting models, the results demonstrate that the prediction error of the varying weights and IOWA operator based combination model is generally smaller. Furthermore, the varying weight combination model exhibits better prediction performance on the test set, resulting in improved prediction accuracy compared to the IOWA operator combination model. These findings suggest that the varying weights and IOWA operator based combination model outperforms other models in predicting compositional data time series. In the future, the application of decomposition method [41] can be considered in the compositional data time series analysis with the combination of the developed model. Besides, robustness of the combination forecasting model is an open and interesting problem. On the other hand, due to the fact of constant sum constraint, a systematic modeling process is needed. Next, how to measure the credibility of CDTSs has not been considered in previous work. Traditional confidence interval method can not be directly used in such field. It’s necessary to developed a parallel or extended theoretical framework to realize the analysis.

## Supporting information

S1 File Compressed/ZIP file codes. (7Z)

## References

[1] Hou G, Song H. Whether the directed technical change promotes the improvement of the energy structure in China. Front Environ Sci. 2022;10. doi: 10.3389/fenvs.2022.928239

[2] Liu Y, Xie X, Wang M. Energy structure and carbon emission: analysis against the background of the current energy crisis in the EU. Energy. 2023;280:128129. doi: 10.1016/j.energy.2023.128129

[3] Jin Y, Wang S, Bu L, Zhai P. Unconventional, conventional monetary policies, and optimal energy supply structure in China. Fin Res Lett. 2023;54:103732. doi: 10.1016/j.frl.2023.103732

[4] Li X, Ge Y, Mao X, Xue W, Xu N. Improved Energy Structure Prediction Model Based on Energy Demand Forecast. In: 2018 2nd IEEE Conference on Energy Internet and Energy System Integration (EI2). 2018. pp. 1–5. 10.1109/EI2.2018.8582170

[5] Vorontsova OV, Ukraintsev VB, Sedykh YA. Innovative Energy Producing Structures in Russia. In: 2019 International Science and Technology Conference “EastConf”. 2019. pp. 1–4. 10.1109/EastConf.2019.8725426

[6] Feng T, Sun L, Zhang Y. The relationship between energy consumption structure, economic structure and energy intensity in China. Energy Policy. 2009;37(12):5475–83. doi: 10.1016/j.enpol.2009.08.008

[7] Yang X, Wang S, Zhang W, Li J, Zou Y. Impacts of energy consumption, energy structure, and treatment technology on SO2 emissions: A multi-scale LMDI decomposition analysis in China. Appl Energy. 2016;184:714–26. doi: 10.1016/j.apenergy.2016.11.013

[8] Jiang P, Yang H, Li H, Wang Y. A developed hybrid forecasting system for energy consumption structure forecasting based on fuzzy time series and information granularity. Energy. 2021;219(C):119599. doi: 10.1016/j.energy.2020.119599

[9] Li H, Li B, Niu D. Prediction on the energy consumption structure in liaoning province based on system dynamics. Pol J Environ Stud. 2021. doi: 10.15244/pjoes/136044

[10] Liu J, Ma H, Wang Q, Tian S, Xu Y, Zhang Y, et al. Optimization of energy consumption structure based on carbon emission reduction target: A case study in Shandong Province, China. Chin J Popul Resourc Environ. 2022;20(2):125–35. doi: 10.1016/j.cjpre.2022.06.003

[11] Guan Y, Yang J, Wang R, Zhang L, Wang M. Exploring the role of energy consumption structure and digital transformation in urban logistics carbon emission efficiency. Atmosphere. 2025;16(8):929. doi: 10.3390/atmos16080929

[12] Martín-Fernández JA, Hron K, Templ M, Filzmoser P, Palarea-Albaladejo J. Model-based replacement of rounded zeros in compositional data: classical and robust approaches. Comput Stat Data Anal. 2012;56(9):2688–704. doi: 10.1016/j.csda.2012.02.012

[13] Kynčlová P, Filzmoser P, Hron K. Modeling compositional time series with vector autoregressive models. J Forecast. 2015;34(4):303–14. doi: 10.1002/for.2336

[14] Snyder RD, Ord JK, Koehler AB, McLaren KR, Beaumont AN. Forecasting compositional time series: a state space approach. Int J Forecast. 2017;33(2):502–12. doi: 10.1016/j.ijforecast.2016.11.008

[15] Liang W, Wu Y, Ma X. Robust sparse precision matrix estimation for high-dimensional compositional data. Stat Probab Lett. 2022;184:109379. doi: 10.1016/j.spl.2022.109379

[16] Tian Y, Majahar Ali MK, Wu L, Li T. Imputation method based on adaptive group lasso for high-dimensional compositional data with missing values. Mal J Fund Appl Sci. 2025;21(1):1551–65. doi: 10.11113/mjfas.v21n1.3621

[17] Qian W, Rolling CA, Cheng G, Yang Y. Combining forecasts for universally optimal performance. Int J Forecast. 2022;38(1):193–208. doi: 10.1016/j.ijforecast.2021.05.004

[18] Kang Y, Cao W, Petropoulos F, Li F. Forecast with forecasts: diversity matters. Eur J Operat Res. 2022;301(1):180–90. doi: 10.1016/j.ejor.2021.10.024

[19] Genre V, Kenny G, Meyler A, Timmermann A. Combining expert forecasts: can anything beat the simple average? Int J Forecast. 2013;29(1):108–21. doi: 10.1016/j.ijforecast.2012.06.004

[20] Yager RR, Filev DP. Induced ordered weighted averaging operators. IEEE Trans Syst Man Cybern B Cybern. 1999;29(2):141–50. doi: 10.1109/3477.752789 18252288

[21] Billheimer D. Compositional Data. Encyclopedia of Environmetrics. 2006.

[22] Kim Y, Heon S, Kenyon J, Kim J, Geller J, Redeker NS. Associations of 24-hour movement behaviors with cardiorespiratory fitness in heart failure patients: a compositional data analysis. Med Sci Sports Exerc. 2025;57(10S):82–82. doi: 10.1249/01.mss.0001155168.29024.c3

[23] Wu Y, Zhang D, Zhang Y, Zhang H, Zhou L, Liu Y, et al. Water wave energy-harvesting accordion structure triboelectric nanogenerators for self-driven corrosion protection. Nano Energy. 2025;142:111207. doi: 10.1016/j.nanoen.2025.111207

[24] Zhang H, Jin J. Assessing the effect of green finance, energy consumption structure and environmental sustainable development: a moderated mediation model. Econ Change Restruct. 2024;57(2). doi: 10.1007/s10644-024-09669-y

[25] Wang H, Liu Q, Mok HMK, Fu L, Tse WM. A hyperspherical transformation forecasting model for compositional data. Eur J Operat Res. 2007;179(2):459–68. doi: 10.1016/j.ejor.2006.03.039

[26] Zhou G, Luo P, He Q. Predicting compositional time series via autoregressive Dirichlet estimation. Sci China Inf Sci. 2018;61(9). doi: 10.1007/s11432-017-9335-5

[27] Barceló-Vidal C, Aguilar L, Martín-Fernández JA. Compositional VARIMA time series. Compositional Data Analysis: Theory and Applications. 2011.

[28] Chang K, Chen R, Fomby TB. Prediction‐based adaptive compositional model for seasonal time series analysis. J Forecast. 2017;36(7):842–53. doi: 10.1002/for.2474

[29] Bates JM, Granger CWJ. The combination of forecasts. J Operat Res Soc. 1969;20(4):451–68. doi: 10.1057/jors.1969.103

[30] Liu J, Wang P, Chen H, Zhu J. A combination forecasting model based on hybrid interval multi-scale decomposition: Application to interval-valued carbon price forecasting. Exp Syst Appl. 2022;191:116267. doi: 10.1016/j.eswa.2021.116267

[31] Radchenko P, Vasnev AL, Wang W. Too similar to combine? On negative weights in forecast combination. Int J Forecast. 2023;39(1):18–38. doi: 10.1016/j.ijforecast.2021.08.002

[32] Pérez-Fernández R, Gagolewski M, De Baets B. On the aggregation of compositional data. Inform Fus. 2021;73:103–10. doi: 10.1016/j.inffus.2021.02.021

[33] Li B, Ding J, Yin Z, Li K, Zhao X, Zhang L. Optimized neural network combined model based on the induced ordered weighted averaging operator for vegetable price forecasting. Exp Syst Appl. 2021;168:114232. doi: 10.1016/j.eswa.2020.114232

[34] Clemen RT. Combining forecasts: a review and annotated bibliography. Int J Forecast. 1989;5(4):559–83. doi: 10.1016/0169-2070(89)90012-5

[35] Plocoste T, Regis S, Nuiro SP, Sankaran A. Application of aggregation operators for forecasting PM10 fluctuations: from available Caribbean data sites to unequipped ones. Atmosph Pollut Res. 2024;15(6):102116.

[36] Huang H, Tian Y, Tao Z. Multi-rule combination prediction of compositional data time series based on multivariate fuzzy time series model and its application. Exp Syst Appl. 2024;238:121966. doi: 10.1016/j.eswa.2023.121966

[37] Lu S, Wang H, Zhao J. Graph convolutional network for compositional data. Inf Fus. 2025;117:102798. doi: 10.1016/j.inffus.2024.102798

[38] Aitchison J. The Statistical Analysis of Compositional Data. London - New York: Chapman and Hall; 1986.

[39] Pérez-Fernández R, Baets BD. Aggregation theory revisited. IEEE Transac Fuzzy Syst. 2021;29(4):797–804. doi: 10.1109/TFUZZ.2020.2965904

[40] Tan F, Wang J, Jiao Y-Y, Ma B, He L. Suitability evaluation of underground space based on finite interval cloud model and genetic algorithm combination weighting. Tunnel Undergr Space Technol. 2021;108:103743. doi: 10.1016/j.tust.2020.103743

[41] Xiao F, Yang S, Li X, Ni J. Branch error reduction criterion-based signal recursive decomposition and its application to wind power generation forecasting. PLoS One. 2024;19(3):e0299955. doi: 10.1371/journal.pone.0299955 38517881 PMC10959340

